# Tularemia — United States, 2001–2010

**Published:** 2013-11-29

**Authors:** Christina Nelson, Kiersten Kugeler, Jeannine Petersen, Paul Mead

**Affiliations:** Div of Vector-Borne Diseases, National Center for Emerging and Zoonotic Infectious Diseases, CDC

Tularemia is a rare but potentially serious bacterial zoonosis that has been reported from all U.S. states except Hawaii. The etiologic agent, *Francisella tularensis*, is highly infectious and can be transmitted through arthropod bites, direct contact with infected animal tissue, inhalation of contaminated aerosols, and ingestion of contaminated food or water (1). *F. tularensis* has been designated a Tier 1 select agent because it meets several criteria, including low infectious dose, ability to infect via aerosol, and a history of being developed as a bioweapon (2). This report summarizes tularemia cases reported to CDC during 2001–2010 via the National Notifiable Diseases Surveillance System (NNDSS) and compares the epidemiology of these cases with those reported during the preceding decade. During 2001–2010, a total of 1,208 cases were reported (median: 126.5 cases per year; range: 90–154). Incidence was highest among children aged 5–9 years and men aged >55 years. Clinicians and public health practitioners should be familiar with the current epidemiology and clinical features of tularemia to identify and adequately treat individual cases and recognize unusual patterns that might signal an outbreak or bioterrorism event.

In humans, *F. tularensis* causes distinct clinical syndromes depending on the route of exposure. Percutaneous inoculation typically produces ulceroglandular tularemia, characterized by a cutaneous ulcer at the site of inoculation and tender regional lymphadenopathy. A less common presentation after percutaneous inoculation is glandular tularemia, in which patients develop regional lymphadenopathy without ulcer. Inhalation of *F. tularensis* can result in a primary pneumonia, whereas ingestion causes oropharyngeal disease consisting of tonsillitis or pharyngitis with cervical lymphadenopathy. Other forms of tularemia include oculoglandular (infection of the eye) and typhoidal (fever without localizing signs) (3). Certain strains of *F. tularensis* subspecies *tularensis* (also known as type A) are associated with more severe disease and a greater risk for death (4,5). Mortality is less than 2% overall but ranges up to 24% depending on the strain (1,4).

For national surveillance purposes, a confirmed case of tularemia is defined as clinically compatible illness with either a four-fold or greater change in serum antibody titer to *F. tularensis* antigen or isolation of *F. tularensis* from a clinical specimen. A probable case is defined as clinically compatible illness with either a single elevated antibody titer to *F. tularensis* antigen or detection of *F. tularensis* in a clinical specimen by fluorescent assay (6). In this report, incidence is calculated using 2005 census population estimates.

A total of 1,208 cases of tularemia were reported via NNDSS during 2001–2010. The median number of cases per year was 126.5, with a range of 90–154 cases per year. Of these 1,208 reported cases, 64% were categorized as confirmed and 35% as probable (Figure 1). Median age of patients was 39 years (range: 1–92 years), and 68% were male. Average annual incidence was 0.041 cases per 100,000 persons. By age group and sex, annual incidence was highest among children aged 5–9 years (0.071) and among men aged 65–69 years (0.11) (Figure 2). Race was recorded for 887 patients (73%). Among these, 86% were white, 9% were American Indian/Alaska Native, and 3% were black. Ethnicity was recorded for 718 patients (59%), of whom 5% were Hispanic. The highest annual incidence by race was among American Indians/Alaska Natives (0.3 per 100,000 persons).

Cases were reported from 47 states (Figure 3). Six states accounted for 59% of reported cases: Missouri (19%), Arkansas (13%), Oklahoma (9%), Massachusetts (7%), South Dakota (5%), and Kansas (5%). Among the 10 states with the highest incidence of tularemia, all but Massachusetts were located in the central or western United States (Table).

Tularemia cases were reported from 505 U.S. counties (16%) during 2001–2010. County of residence was available for 1,198 patients (99%), although in some cases this might not have been the county of exposure. Among these, 53% of patients resided in counties classified as rural by CDC National Center for Health Statistics’ *Urban-Rural Classification Scheme for Counties* (7), although rural counties accounted for only 17% of the U.S. population in 2006. The county with the highest annual incidence was Dukes County (Martha’s Vineyard and the Elizabeth Islands), Massachusetts (67 cases; 43 per 100,000 persons). Cases in Dukes County were reported consistently during the 10-year period (range: 2–16 cases per year), with substantial increases in 2005 (11 cases), 2006 (10 cases), and 2008 (16 cases). Additional counties with high incidence rates were Buffalo County, South Dakota (six cases; 29 per 100,000), and Shannon County, South Dakota (24 cases; 18 per 100,000).

The majority of cases (77%) occurred during May through September, consistent with peak arthropod activity and increased outdoor human activity. However, seasonal patterns varied by region. In the New England states, no cases occurred in the nonpeak winter months of December through March. In contrast, 20% of cases in the South Atlantic states, 15% in the East South Central states, and 14% in the Pacific states occurred from December through March.

The total number of cases reported during 2001–2010 was similar to the number reported during the 10-year period 1991–2000 (1,208 versus 1,216, respectively). Nevertheless, notable changes occurred in the number of cases reported from some individual states: Montana (72% decrease), Arkansas (42% decrease), South Dakota (29% decrease), Massachusetts (155% increase), Nebraska (120% increase), and Oklahoma (35% increase) (8).

## Editorial Note

During 2001–2010, the number and demographic features of reported tularemia cases were similar to those reported during the preceding decade. Nevertheless, several differences were noted between the two periods. The geographic distribution of reported cases was slightly less concentrated in the central states during 2001–2010, with a greater proportion of cases reported from the Northeast and the Pacific states of Washington and California than in previous years. In addition, four states that had not reported cases during 1991–2000 (Connecticut, New Hampshire, Vermont, and West Virginia) reported cases during 2001–2010. Further investigation is needed to determine whether the change in distribution was caused by alterations in reporting patterns, vector distribution, human behavior, or other factors.

Seasonal variations by region are likely attributable in part to climate differences, because states with milder climates have longer arthropod activity and thus extended periods of risk. Seasonal variations might also reflect, to some extent, hunting activities that can occur year-round, in contrast to landscaping and other outdoor recreational activities that are concentrated in the summer months. Hunting can result in human exposure to tularemia through direct contact with infected animals and ingestion of infected meat. Hunting of rabbits, which typically occurs in the fall and winter, might explain the higher proportion of winter cases in South Atlantic and East South Central states, where small game hunting is common (9).

In a previous surveillance report for the period 1990–2000, CDC recommended improving surveillance by increasing documentation of laboratory confirmation and collecting more detailed epidemiologic and clinical data (8). Documentation of laboratory confirmation has indeed improved; during 1990–2000, only 65% of case reports included documentation indicating whether they met the probable versus confirmed case definition, compared with 99% during 2001–2010. Although the amount of epidemiologic and clinical data collected through NNDSS has not changed, CDC does regularly request additional patient information from health departments to better characterize the disease.

The findings in this report are subject to at least two limitations. First, the tularemia cases described in this report might not be fully representative of all cases diagnosed in the United States because case ascertainment and reporting might be incomplete and differ by state. Second, missing data and small numbers limit statistical comparisons and interpretation.

Tularemia is not a common disease, but it continues to cause approximately 100 reported human cases annually in the United States and is a serious and potentially fatal disease. Although outbreaks do occur (10), the majority of reported tularemia cases in the United States are sporadic. Clinicians should consider tularemia in patients with a compatible clinical profile, particularly in children and elderly males with acute fever and regional lymphadenopathy. This report shows that the distribution of tularemia might be gradually changing; therefore, tularemia should be considered even in areas where it has rarely been reported.

What is already known on this topic?

Tularemia is a bacterial zoonosis that can be acquired through various exposure routes. It has been reported in every state except Hawaii, but cases most commonly occur in central U.S. states. It is caused by *Francisella tularensis*, an organism classified as a Tier 1 select agent based on its potential use for bioterrorism.

What is added by this report?

The total number of tularemia cases reported via the National Notifiable Diseases Surveillance System during 2001–2010 did not differ from the number reported during the preceding decade. Geographic distribution was less centrally concentrated; 66% of northeastern states and two Pacific states reported more cases during 2001–2010 compared with 1991–2000. Although the majority of patients with tularemia (53%) resided in rural counties, a substantial proportion of patients (47%) resided in urban counties.

What are the implications for public health practice?

Although aspects of tularemia surveillance have improved, such as documentation of laboratory confirmation, underreporting and other limitations remain. It is important to maintain and enhance surveillance and collect detailed clinical information on each case to enhance understanding of the disease and elucidate the causes of recent epidemiologic shifts.

State and local public health departments are encouraged to report tularemia cases in a timely manner and provide additional patient information, including exposure history, clinical syndrome, and outcome, to CDC when possible. Because the threat of bioterrorism remains, clinicians and health departments should remain vigilant; for example, an urban cluster of tularemia cases among persons without a common natural exposure could be the first sign of a bioterrorism attack.

## Figures and Tables

**FIGURE 1:**
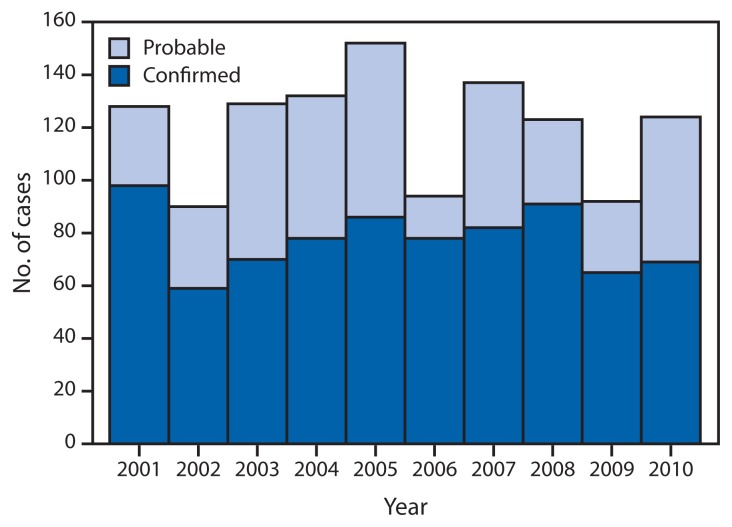
Number of reported cases of tularemia, by case status and year — United States, 2001–2010

**FIGURE 2:**
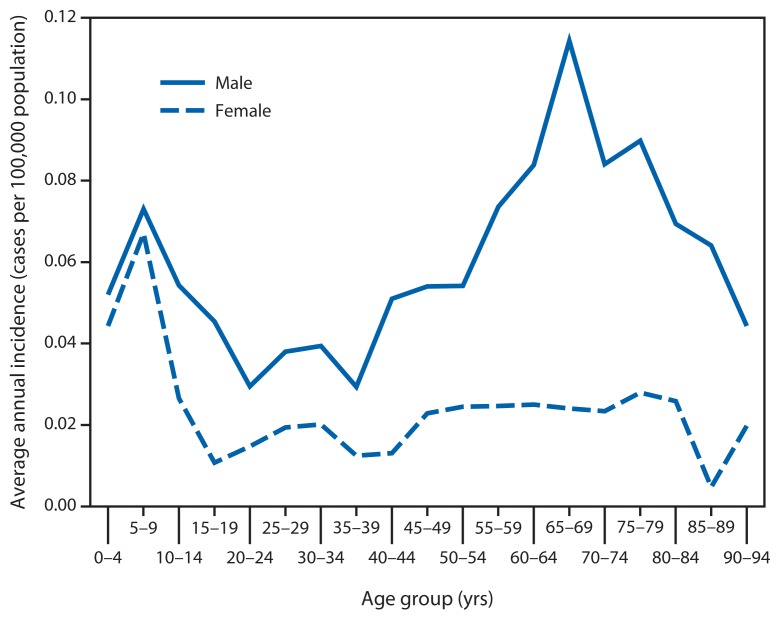
Average annual incidence of tularemia, by age group and sex — United States, 2001–2010

**FIGURE 3:**
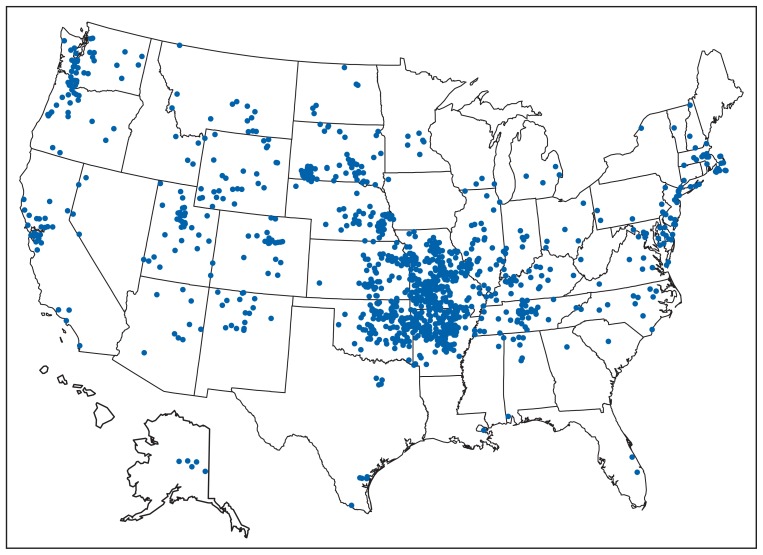
Reported cases of tularemia — United States, 2001–2010* * One dot is placed randomly within county of residence for each reported case.

**TABLE:** Ten states with the highest incidence of tularemia — United States, 2001–2010

State	Total no. of reported cases	Incidence*
South Dakota	65	0.84
Arkansas	162	0.58
Wyoming	29	0.57
Missouri	231	0.40
Nebraska	55	0.31
Oklahoma	108	0.30
Kansas	59	0.22
Montana	13	0.14
Massachusetts	84	0.13
Utah	32	0.13

* Incidence calculated as reported cases per 100,000 persons per year.
